# Phenylacetic Acid Is ISR Determinant Produced by *Bacillus fortis* IAGS162, Which Involves Extensive Re-modulation in Metabolomics of Tomato to Protect against *Fusarium* Wilt

**DOI:** 10.3389/fpls.2016.00498

**Published:** 2016-04-19

**Authors:** Waheed Akram, Tehmina Anjum, Basharat Ali

**Affiliations:** ^1^Institute of Molecular Biology and Biotechnology, University of LahoreLahore, Pakistan; ^2^Institute of Agricultural Sciences, University of the PunjabLahore, Pakistan; ^3^Department of Microbiology and Molecular Genetics, University of the PunjabLahore, Pakistan

**Keywords:** induced systemic resistance (ISR), *Bacillus*, tomato, *fusarium* wilt, phenylacetic acid

## Abstract

*Bacillus fortis* IAGS162 has been previously shown to induce systemic resistance in tomato plants against *Fusarium* wilt disease. In the first phase of current study, the ISR determinant was isolated from extracellular metabolites of this bacterium. ISR bioassays combined with solvent extraction, column chromatography and GC/MS analysis proved that phenylacetic acid (PAA) was the potential ISR determinant that significantly ameliorated *Fusarium* wilt disease of tomato at concentrations of 0.1 and 1 mM. In the second phase, the biochemical basis of the induced systemic resistance (ISR) under influence of PAA was elucidated by performing non-targeted whole metabolomics through GC/MS analysis. Tomato plants were treated with PAA and fungal pathogen in various combinations. Exposure to PAA and subsequent pathogen challenge extensively re-modulated tomato metabolic networks along with defense related pathways. In addition, various phenylpropanoid precursors were significantly up-regulated in treatments receiving PAA. This work suggests that ISR elicitor released from *B. fortis* IAGS162 contributes to resistance against fungal pathogens through dynamic reprogramming of plant pathways that are functionally correlated with defense responses.

## Introduction

During the long history of coevolution between host plants and pathogens, plants have developed their own strategies to combat with pathogens. Plant interactions with other organisms lead to cross-talk between signaling pathways that helps to activate different defense responses against pathogens (Beckers and Spoel, 2006). These interactions can eventually cross the border between the aerial parts of plants and the roots (Garcia-Brugger et al., 2006; Pieterse et al., 2009; van Dam, 2009). In case of plant-pathogen interactions, plants can produce immune signals from the infection sites (Ausubel, 2005; Jones and Dangl, 2006). These immune signals activate batteries of defense responses (Chisholm et al., 2006; Dodds and Rathjen, 2010).

Induced systemic resistance (ISR) is a great regulatory potential of plants that activates appropriate cellular defense responses before or upon pathogen attack. ISR activates plant immunity in a similar way as induced by pathogens or insects that are specifically directed against invaders in incompatible interactions (Conrath et al., 2002). ISR is accompanied with augmented expression of defense related genes, increased accumulation of secondary metabolites, and defense associated proteins (van Loon et al., 1998; Conrath, 2006; van Hulten et al., 2006; Zamioudis and Pieterse, 2012). These induced defense mechanisms are dependent on jasmonic acid (JA) and ethylene (ET) signaling in the plants (van Loon et al., 1998; Conrath et al., 2006). In addition, these altered traits directly and indirectly influence defense related mechanisms of host plants and their growth attributes. Finally, plants defend themselves against invading pathogens through a combination of induced and constitutive defenses that negatively affect pathogen performance. Another type of resistance mechanism in plants is termed as “Systemic acquired resistance” (SAR). SAR is associated with the perception of elicitors from avirulent pathogens (Thakur and Sohal, 2013). SAR is phenotypically similar to ISR, which is also effective against diverse pathogens. SAR is dependent on salicylic acid (SA) signaling pathway (Park et al., 2008) while ISR typically relies on the JA and ET signaling pathways (Pieterse et al., 2002).

Plant responses to infectious agents are mediated by recognition of microbial signaling molecules (Gómez-Vásquez et al., 2004; Garcia-Brugger et al., 2006; Conrath, 2011). These microbial signaling molecules associated with some beneficial microbes, are termed as microbe-associated molecular patterns (MAMPs) (Boller and Felix, 2009; Mishra et al., 2009). These MAMPs upon recognition initiate basal defense responses throughout the plant body (Montesano et al., 2003; Ryan and Pearce, 2003; Gómez-Vásquez et al., 2004). MAMPs mediated ISR is not related with direct activation of plant defense related genes, rather it implies quicker and stronger activation of some basal plant defense responses upon attack of a pathogen (Zipfel et al., 2004, 2006; Mishra et al., 2009). This MAMPs based resistance is effective enough to hinder infection and pathogen establishment inside plant body (Katagiri and Tsuda, 2010). Plant growth-promoting rhizobacteria can elicit ISR in plants by secreting MAMPs, which are also termed as ISR elicitors or determinants (van Loon et al., 1998; Bakker et al., 2003; Persello-Cartieaux et al., 2003; Lugtenberg and Kamilova, 2009). Along with MAMPs, recently some phytohormones have been found capable to elicit induced resistance in some plants. Some studies have revealed new insights into the role of auxins in plant defense (Kazan and Manners, 2009). Similar to the application of the defense-eliciting hormones, SA and JA, exogenous application of auxins can positively affect resistance against some pathogens (Sharaf and Farrag, 2004).

Several studies concerning the screening of bacterial strains capable to elicit ISR in plants have been conducted, yet relatively few studies have focused on the recognition of MAMPs produced by these bacteria and dynamic changes of metabolic responses in plants under influence of these MAMPs. Some secondary metabolites produced by bacteria have been recognized as ISR elicitors. These include as 4-aminocarbonyl phenylacetate secreted by *Klebsiella oxytoca* C1036 (Park et al., 2009), *N*-alkylated benzylamine derivative produced by *Pseudomonas putida* BTP1 (Ongena et al., 2005) and dimethyl disulfide produced extracellularly by *Bacillus cereus* C1L (Huang et al., 2012). Some bacteria produce volatile organic compounds as 2-3-butanediol and acetoin, which can trigger ISR in host plants upon recognition (Ryu et al., 2004).

Metabolomics is a promising analytical technology that has been used to unravel the metabolic fluctuations of plant molecules and the re-programing associated with various plant pathways (De Vos et al., 2007; Zhao et al., 2015). This technique is considered one of the most rapidly growing areas of modern science. The transcriptional or protein profile cannot be directly linked with metabolic changes (Zhao et al., 2015). We previously proved that *B. fortis* IAGS162 is a beneficial rhizospheric bacterium capable of managing *fusarium* wilt disease of tomato by ISR phenomenon in tomato plants (Akram et al., 2013). This study reports phenylacetic acid (PAA) as an ISR elicitor that is secreted extracellularly by this beneficial bacterial strain. It also describes significant metabolic rerouting in the plant pathways under influence of this ISR elicitor. Moreover the compatible host pathogen interactions have been characterized by a lower level of certain defense-related mechanisms compared with host pathogen interactions in the presence of ISR elicitor that leads to a more dynamic metabolic response over the course of colonization. We have also discussed the potential relevance of these changes in host defense responses.

## Methodologies

### Microbial Treatments Preparation

The tomato wilt pathogen “*F. oxysporum* f.sp. lycopersici” was provided by Fungal biotechnology lab, Institute of Agricultural sciences, University of the Punjab, Lahore. This fungus was grown on potato dextrose agar (PDA Difco) for 1 week. Conidia were harvested by scraping and conidial suspension was prepared in sterilized water at a concentration of 10^5^ conidia/ml. ISR capable bacterial strain *Bacillus fortis* IAGS162 (Akram et al., 2013) was cultured in Luria Bertani (LB) broth media overnight at 35°C. Cell free culture filtrate (CFCF) was obtained by centrifugation at 4000 *g* for 20 min and used for ISR assay. Bacterial pallet was re-suspended in sterilized distilled water to a final concentration of 10^4^ cfu/ml for application. Intracellular metabolites were extracted by sonicating the bacterial cell suspension at resonance amplitude for 30 s to obtain intra-cellular components.

### Preliminary Screening of ISR Determinants from *B. fortis* IAGS162

This experiment was performed for preliminary screening of ISR determinant/s either present in intracellular metabolites or CFCF of *B. fortis* IAGS162 against *fusarium* wilt, as previously described by Akram et al. (2015). Briefly, tomato plants of *fusarium* susceptible variety were raised from sterilized seeds, inside plastic pots containing sterilized commercial potting mix. Fourteen days after emergence, tomato plants were treated with 50 ml of intra-cellular metabolites and CFCF of *B. fortis* IAGS162 separately. Here plants in positive control were provided with 50 ml of a water based formulation of *B. fortis* IAGS162, while the water treated control plants got 50 ml of distilled water. Three days after treatments with abovementioned substances, the wilt pathogen was provided by adding 50 ml of the *F. oxysporum* conidial suspension. The pots were incubated in a greenhouse for 15 days under natural day light conditions. Each treatment had five replicate plants, and experiment was performed twice. To determine the DI, wilting was scored based on the criteria developed by Epp (1987). The equation described by Cachinero et al. (2002) was used to calculate the DI.

(1) DI=[(Σni×si)/(N×S)×100]

where, ni = the number of diseased plants, si = value of the disease score, N = the total number of plants observed, and S = maximum rank of disease score.

### Isolation of ISR Determinants from CFCF of *B. fortis* IAGS162

**FIGURE 1:**
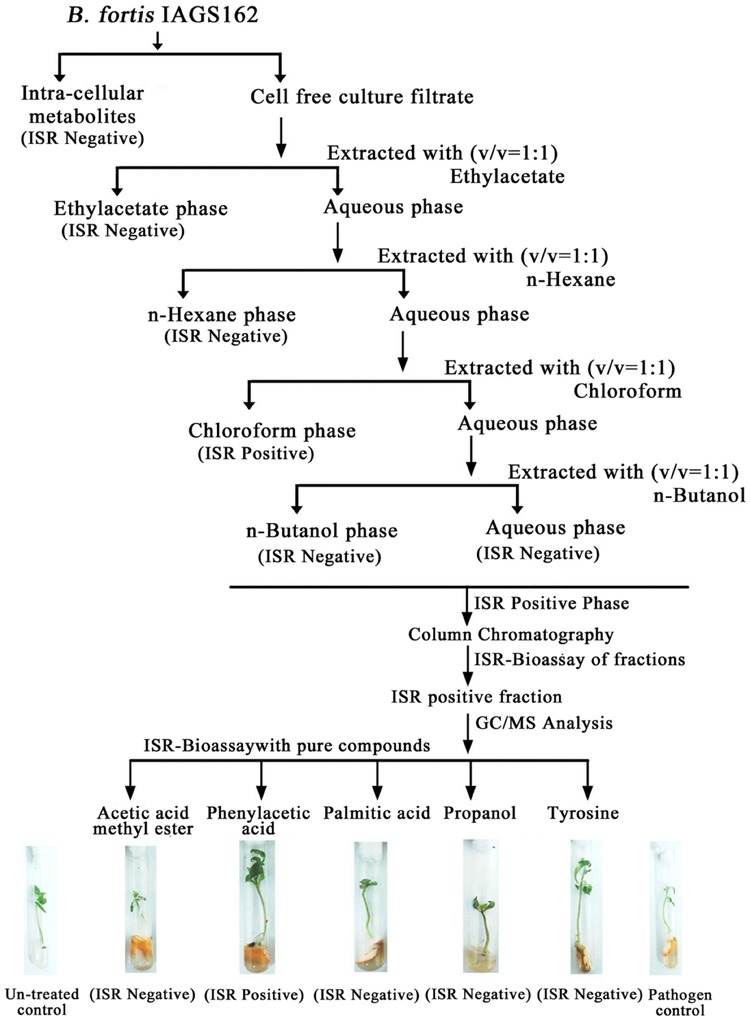
**Extraction procedure and ISR activity of crude and purified metabolites from *B. fortis* IAGS162**.

Cell free culture filtrate of *B. fortis* IAGS162 was prepared as described previously and extracted twice with series of organic solvents (**Figure 1**). All the organic extracts were thoroughly dried, then dissolved in 10% dimethyl sulfoxide (DMSO). These were amended in Murashige and Skoog (MS) medium at a concentration of 0.1%, and ISR bioassay was performed as described below. The extract with the positive ISR activity was partitioned into sub-fractions by performing silica gel column chromatography by stepwise elution method by using methanol and ethyl acetate. These sub-fractions were dissolved in 10% DMSO after drying, and 10 μl of each DMSO based formulation was again used in ISR bioassay.

The sub-fraction showing ISR activity was subjected to GC/MS analysis as described by Akram et al. (2015). The compounds identified by GC/MS analysis, present in the ISR-active sub-fraction were purchased from Sigma–Aldrich. ISR bioassay was again performed by adding these pure compounds in MS media at three varying concentrations viz: 0.01, 0.1, and 1 mM.

### ISR Bioassay

For ISR bioassays and metabolomic analysis, tomato plants of *fusarium* susceptible variety were grown in MS medium inside glass culture tubes in a growth chamber for seedling development (25°C, 16 h light). At the age of 5 days, growth media was added with different elicitor preparations that were to be tested for ISR activity. Two days after elicitor application, tomato plants were treated by adding 10 μL of the pathogen inoculum in the form of an aqueous spore suspension at a concentration of 10^5^ conidia/ml. Tomato plants were incubated under same conditions as indicated above. DI was recorded after 1 week of incubation, as described earlier. The experiment included 10 replicates of each treatment and was repeated twice.

### Tomato Metabolome Analysis for Elucidation of PAA Mediated ISR Mechanism

Another independent test tube bioassay was performed to elucidate PAA mediate ISR process in tomato plants against *fusarium* wilt disease as described above. This experiment included four treatments: untreated control plants, pathogen challenged plants, PAA (0.01 mM) treated plants, PAA (0.01 mM) + pathogen treated plants. After 1 week of treatment applications, shoot samples of plants were taken for GC/MS analysis. The metabolite extraction process was carried out by using a single-phase solvent (chloroform/methanol/water). This solvent system has been developed to recover a wide range of metabolites (Catchpole et al., 2005; Beckmann et al., 2007). Three replicate shoot samples, obtained from five plants of two independent experiments, were used in this GC-MS analysis.

### Metabolomic Analysis

Methodology proposed by Lisec et al. (2006) was used to perform tomato metabolome analysis. Metabolites were identified by comparing their spectral similarity in the NIST library. Metabolite levels were determined using the Mzmine software package^1^. Obtained values were log10-transformed (Steinfath et al., 2008) and normalized to show identical medium peak sizes per sample group. Statistical analyses and graphical representations were performed using DSAASTAT and the ClustVis: a web based multivariate data analysis tool. The PCA analysis was performed using the ‘bpca’ algorithm in ClustVis online tool^2^. The metabolite data were summarized using the heatmap function in ClustVis tool with row wise scaling and correlation-based clustering.

### Statistical Analysis

The data were analyzed by performing analysis of variance (ANOVA), and the significance of treatment was determined by Duncan’s new multiple range test (DNMRT) at *P* ≤ 0.05 with the software DSAASTAT (Onofri Italy).

## Results

### Preliminary Screening of ISR Determinants from *B. fortis* IAGS162

In this study, we tested mainly the control efficacy of intracellular metabolites and CFCF of *B. fortis* IAGS162 against *fusarium* wilt of tomato in greenhouse. Data regarding disease index showed a significant response of different treatments on plant survival. Both CFCF and alive cells of *B. fortis* IAGS162 (used as a positive control) displayed efficacy in control of *fusarium* wilt disease (**Table 1**). Indeed, treatment with CFCF was as effective as the alive bacterial cells and resulted in approximately 70% reduction in disease index as compared to the pathogen control. In contrast the plants treated with intracellular metabolites exhibited no considerable protection against *fusarium* wilt disease (**Table 1**). Thus, these results suggested that CFCF from *B. fortis* IAGS162 could be mainly responsible for the suppression of *fusarium* wilt disease and carry the potential ISR determinant/s.

**Table 1:** Potential of *B. fortis* IAGS162 and its components to induce systemic resistance in tomato against *fusarium* wilt.

Treatments	Disease index (DI)
Alive cells	22.18 ± 03.82^D^
Heat killed cells	81.06 ± 11.26^AB^
Intra-cellular components	78.92 ± 06.91^B^
CFCF	34.38 ± 02.08^C^
Pathogen control	86.53 ± 11.47^A^
Non-treated Control	–

*CFCF, cell free culture filtrates. Numbers with ± represent Standard error between different replicates of same treatments. Different letters shows levels of significance as governed by ANOVA and DNMRT at P ≤ 0.05.*

### Isolation of ISR Determinants from CFCF of *B. fortis* IAGS162

**FIGURE 2:**
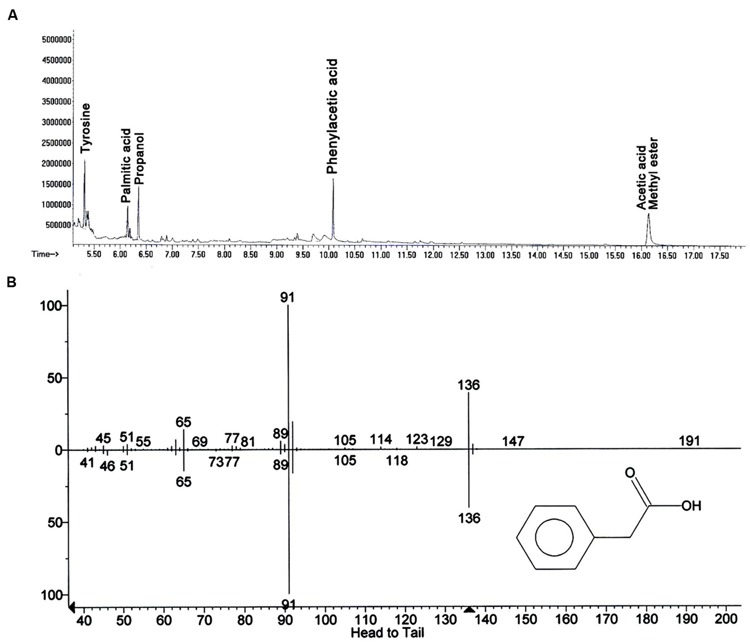
**Elucidation of biochemicals presents in ISR active sub-fraction in cell free culture filtrates of *B. fortis* IAGS162. (A)** Chromatogram of Gas Chromatography and Mass Spectrometry analysis (GC/MS) for identification of the ISR determinant/s of *B. fortis* IAGS162 present in the ISR-active sub-fraction. **(B)** The mass spectrum analysis obtained by electrospray ionization of Phenylacetic acid.

In search for the ISR determinant/s, CFCF of *B. fortis* IAGS162 was extracted by using organic solvents system and ISR bioassays were performed by using extracts obtained. Compared to the rest of the treatments, chloroform fraction elicited ISR in tomato plants against *fusarium* wilt disease (**Figure 1**). Biochemcials present in this fraction were further sub-divided by performing silica gel chromatography, and the sub-fractions were again used in ISR bioassays. GC/MS analysis identified five compounds in ISR active sub-fraction including, acetic acid methyl ester, PAA, palmitic acid, propanol, and tyrosine (**Figure 2**).

**FIGURE 3:**
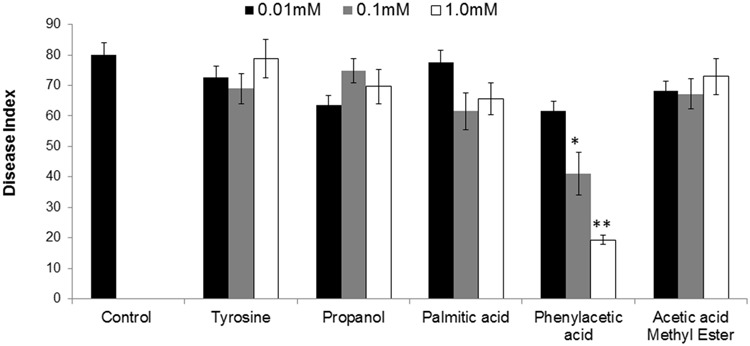
**Influence of root treatment with chemicals present in ISR active sub-fraction on the disease development on tomato plants after inoculation with *fusarium* wilt pathogen.** Sterile distilled water was used as positive control. ISR eliciting sub-fraction was subjected to GC/MS analysis and chemicals present were purchased and subjected to ISR bioassay. Vertical bar represents standard errors. Asterisks indicate statistically significant reduction in disease index as compared to pathogen control as governed by ANOVA at *P* ≤ 0.05.

To finally confirm the potential of ISR determinant/s, use of pure biochemicals as present in ISR active sub-fraction was focused upon. In these ISR bioassays, only PAA was found to suppress *fusarium* wilt (**Figures 1** and **3**). Thus the results indicated that 0.1 and 1.0 mM PAA significantly (*P* ≤ 0.05) reduced disease index up to 58.4 and 76.7%, respectively, as compared to that in the pathogen control (**Figure 3**). The other compounds failed to elicit significant (*P* ≤ 0.05) reduction in DI (**Figures 1** and **3**). Conspicuously, tomato plants receiving PAA provided better growth as compared to the rest of the treatments in this ISR bioassay that can be attributed toward plant growth promoting capability of PAA (**Figure 1**).

### GC/MS Analysis of Tomato Metabolome

**FIGURE 4:**
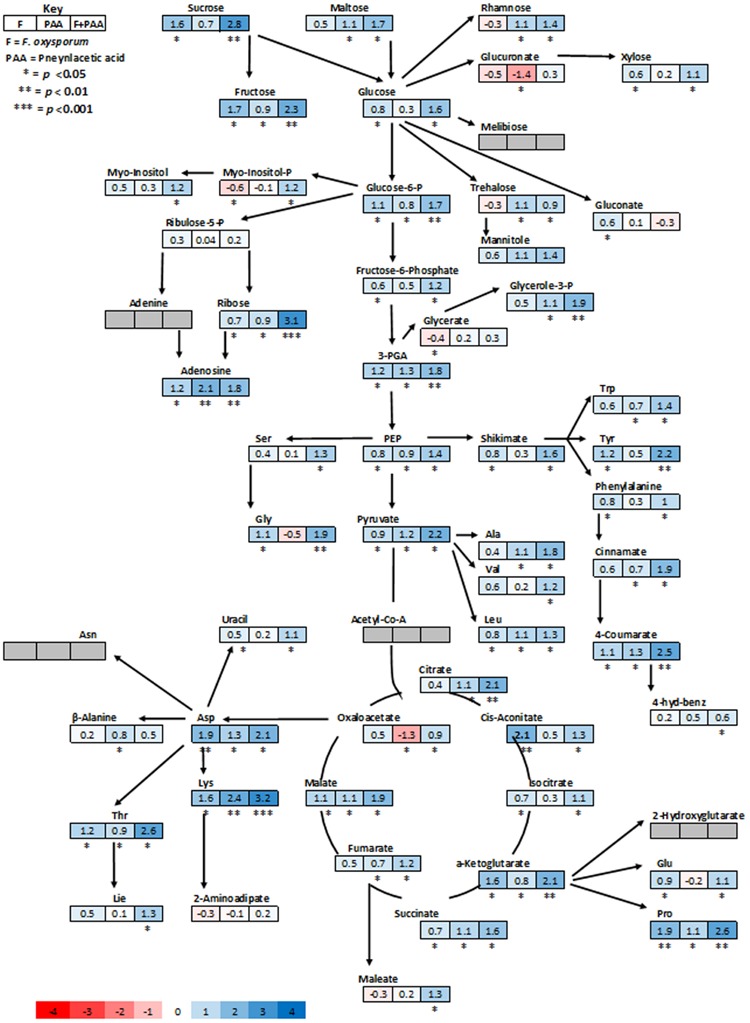
**Schematic representations of tomato metabolites in fold change as compared to untreated control plants.** Different colors represent levels of metabolite fold change where blue is increasing and red is decreasing. Mean values of two independent experiments are presented here.

Metabolites are considered as signaling molecules as they are associated with physiological processes. To elucidate ISR process that may be involved in PAA-mediated resistance to *fusarium* wilt disease, we analyzed the whole metabolome of tomato plants inoculated with the pathogen and PAA in either combination. Central metabolites changed in response to PAA + *F. oxysporum*, PAA, and *F. oxysporum* alone were normalized to respective control and were expressed in fold change (**Figure 4**). Here we identified more than 60 metabolites whose levels were seemed to be altered in response to different treatments (**Figure 7**).

**FIGURE 5:**
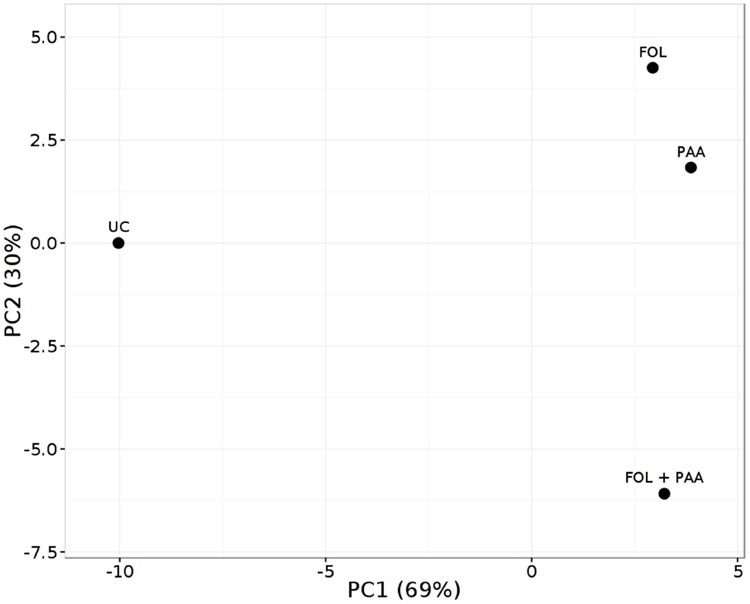
**Principal component analysis (PCA) score plot of metabolite finger printing of tomato shoots 7 days after treatment applications.** FOL, *F. oxysporum* f.sp. lycopersici. PAA, phenylacetic acid. UC, non-treated control.

As expected, the most represented categories included metabolites involved in defense pathways, metabolites involved in cell communication and signaling, and metabolites implicated in primary and secondary metabolism. The PCA scores plot revealed a clear separation of the all different treatments and demonstrating the significant and differential effect of the treatments on the metabolic level (**Figure 5**). Quantities of more than 40 metabolites appeared significantly increased or repressed in treatments receiving PAA in either combinations as compared to rest of the treatments.

**FIGURE 6:**
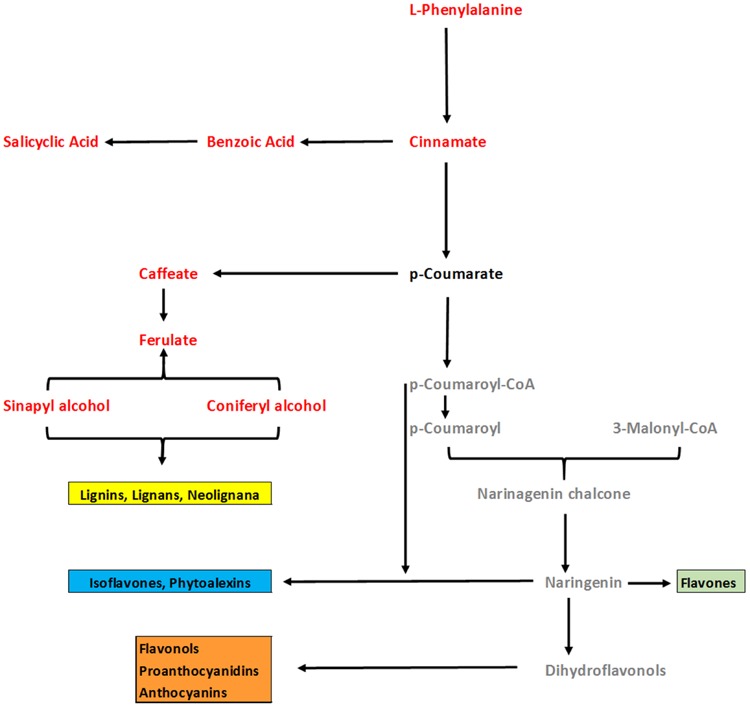
**An overview of changes in phenylpropenoid pathway of tomato plants under influence of PAA and *fusarium* wilt pathogen.** Comparison was made between two treatments viz: 1 = Plants receiving PAA and *F. oxysporum*, 2 = plants receiving *F. oxysporum* alone (considered as control). Metabolites in red font color show significant increase over control as governed by ANOVA at *P* ≤ 0.05. Black font represent no significant change as compared to control. Gray color represent metabolites not detected.

In general, analysis of tomato plants challenged by PAA and *F. oxysporum* showed that the primary metabolism was significantly reprogrammed in both cases but with different consequences (**Figure 4**). Number of metabolites involved in the shikimate and the phenylpropanoid pathways were up-regulated under influence of either pathogen alone on in combination to PAA as compared to the control plants (**Figures 4** and **6**). However, some metabolites specifically precursors of phenylpropanoid pathway were not significantly up-regulated by pathogen alone viz: (tryptophan, cinnamic acid, 4-hydroxybenzene) although they were induced by PAA in combination to the pathogen (**Figures 4** and **6**).

**FIGURE 7:**
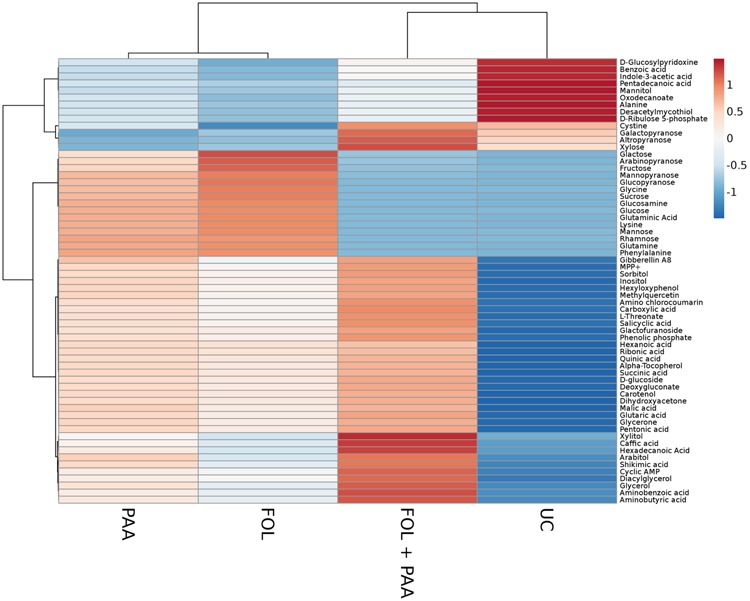
**Heat map illustrating the different metabolite levels in tomato plants under different treatments.** Each row represents differentially expressed metabolites while each column represents a treatment. Mean values of two independent experiments are presented here. Metabolites are clustered using average linkage hierarchical clustering. The colors in the heat map represent the intensity of the log^2^-fold change in metabolite levels. FOL, *F. oxysporum* f.sp. lycopersici. PAA, phenylacetic acid. UC, non-treated control.

In accordance with these observations, plants treated with pathogen alone showed a significant down-regulation of some glycolytic, amino acid metabolism and TCA pathway intermediates viz: (Glucoronate, Myo-inositol, treahlose, 2-aminoadipitate and malic acid) but an increase was observed under synergistic effect of PAA and *F. oxysporym* (**Figure 4**). In the same way, in tomato plants primed with PAA in either combination, the glycolytic and TCA pathways were up-regulated, and their precursors were also significantly increased. This may be related to the different elicitation and signaling progression between the two elicitors (PAA and *F. oxysporum*) (**Figure 4**). Along with that, some resemblance was also observed between PAA mediated ISR and pathogen triggered SAR responses. It gives us clue that PAA-mediated activation and up-regulations of metabolites may contribute to PAA-induced resistance against *fusarium* wilt disease.

## Discussion

### Searching ISR Determinant/s from *B. fortis* IAGS162

The potential importance of a beneficial rhizospheric bacterial strain was first reported when author observed ISR eliciting capability of *B. fortis* IAGS162 against *fusarium* wilt of tomato (Akram et al., 2013). Beside a limited number of researches screening the potential MAMPs from these beneficial symbiotic microbes, a lot of studies to date appears to describe screening the beneficial microbes. However, there do not exist follow up studies that elucidate detailed mechanisms behind MAMPs mediated ISR in crop plants against diseases. Initial studies were conducted to search the ISR determinant/s responsible for elicitation of ISR in tomato against *fusarium* wilt disease. In preliminary experiments, CFCF significantly reduced the disease effect, clued that ISR determinant/s resides in CFCF of *B. fortis* IAGS162. The largest significant reduction in disease index was obtained by CFCF treatment (**Table 1**). The ISR elicitors from beneficial microbes can be either produced in intracellular matrix or secreted extracellularly in the rhizosphere. In some studies, it was shown that ISR determinant/s were retained in CFCF of some microbes (Van Peer and Schippers, 1992; Leeman et al., 1996; Gomez-Gomez and Boller, 2002). Here chloroform phase of culture filtrates of *B. fortis* IAGS162, was containing ISR elicitor as directed by ISR bioassays. The metabolites present in this phase were further separated into sub-fractions and subjected to GC/MS analysis. Further ISR bioassays guided that among different compounds present in ISR sub-fractions, only PAA effectively switched on systemic resistance in tomato plants against *fusarium* wilt disease (**Figure 4**).

Auxins have long been recognized as regulators of plant growth, but some recent studies have discovered the role of auxins in plant defense mechanisms. Some phytohormones including auxins have recently emerged as re-modulators of plant defense against pathogens (Robert-Seilaniantz et al., 2011). Recent advances in plant immunity suggest that auxins and JA act synergistically and their signaling pathways share many commonalities (Kazan and Manners, 2009). In *Arabidopsis*, jasmonate (JA) signaling repressor has been shown to be stimulated by auxins (Grunewald et al., 2009). Auxins and some defense related phytoalexins are synthesized by the same tryptophan pathway (Tan et al., 2007). PAA, also known as benzeneacetic acid, is an aromatic carboxylic acid having a phenyl functional group. It is also a naturally occurring auxin that is present in many plant species (Abe et al., 1974; Schneider and Wightman, 1986) and produced by some bacteria (Sarwar and Franckenberger, 1995; Hwang et al., 2001; Kim et al., 2004: Somers et al., 2005; Sumayo et al., 2013). In this study, PAA effectively elicited ISR in tomato plants when applied at concentrations of 0.1 and 1 mM. In another study, this kind of ISR was elicited by PAA along with 1-hexadecene and linolic acid secreted by *Ochrobactrum lupini* KUDC1013 (Sumayo et al., 2013). In the same way, rhizospheric application of an auxin (IAA) trigerred ISR in tomato plants against *fusarium* wilt of tomato along with positive effect on growth and fruit yield (Sharaf and Farrag, 2004).

### Elucidation of Mechanism behind PAA Induced ISR in Tomato

Metabolites are the signaling molecules, involved in both constitutive and inducible plant defense responses that interfere negatively with pathogen performance. GC/MS is an analytical technique in metabolomics for detection, identification, and analysis of small molecules (Budak et al., 2015). Plants accumulate some metabolites to protect them from both biotic and abiotic stress for their survival. First phase of this study suggested that PAA is an ISR determinant produced by *B. fortis* IAGS162. However, a conclusive demonstration that PAA mediated ISR elicitation activity leads to significant increase in levels of plant metabolites during infection process was still lacking. Hence it was required to elucidate its role in basal resistance to pathogens. For that purpose, we carried out a complete metabolome analysis of tomato plants treated with PAA and infected with *F. oxysporum* to determine what responses activated by PAA either alone or in combination to pathogen, may be involved in tomato defense against the fungal pathogen *F. oxysporum.* The metabolites induced by both PAA and *F. oxysporum* infection not only encode defense-related biochemicals (phytoalexins), but also other biochemicals involved in primary and secondary metabolisms.

In our study, PAA application in either combination caused significant increase in some sugar contents viz: sucrose, maltose, glucose and fructose. Carbohydrates are considered the major source of energy for the metabolic changes that occur in plants. Their availability is of major relevance to the plant growth and might be associated with the induction of defense responses in plants. This increase in sugar contents might lead to increased biosynthesis of numerous precursors involved in other secondary metabolic pathways. Furthermore, increased sugars production has been speculated to provide the phosphate sugars that are used for antioxidant pathway activity and phenolic synthesis (Shetty and Wahlqvist, 2004). We also observed dynamic changes in polyols such as myo-inositol, under influence of PAA in tomato plants. Accumulation of polyols is reported to be associated with stress tolerance in plants (Stoop et al., 1996).

Phenylpropanoids are secondary metabolites that play a basic role in signaling and plant defense responses against abiotic or biotic constraints (Nugroho, 2002; Reuben et al., 2013). GC/MS analysis confirmed that some precursor compounds of phenylpropenoid pathway like L-phenylalanine, cinnamic acid, benzoic acid, caffeic acid, and salicyclic acid, were distinctively increased in tomato plants receiving PAA. L-phenylalanine is a precursor of a wide range of natural products. Enzymatic deamination of L-phenylalanine directs the energy flow to the various branches of the general phenypropanoid metabolism (Vogt, 2010). Along with that, inoculation of tomato plants with PAA led to increased synthesis of benzoic acid, a precursor of salicyclic acid biosynthesis. Salicyclic acid is a key signaling compound triggering local and systemic resistance in plants against both biotic and abiotic stresses (Shah and Klessig, 1999). Caffeic acid is mainly involved in lignification of plant cell walls and may have other physiological functions (Rastogi and Dwivedi, 2008). These results suggest that up-regulation of these compounds are likely to re-enforce plant defense responses against invading pathogen.

Phenylpropanoid-based polymers in plants, such as lignin, suberin, or condensed tannins, contribute to the physical stability and robustness toward environmental damages from drought or wounding (Conrath et al., 2001; Anterola and Lewis, 2002; Reuben et al., 2013). In accordance with the results of GC/MS analysis, an overview of PAA-modulated changes in quantities of precursors involved in the phenylpropanoid pathway showed that this pathway is clearly involved in ISR, leading to the production of phytoalexins such as lignin, flavonoids and anthocyanins. Tomato plants treated with PAA in combination with the pathogen challenge, provided a maximum increase in the levels of defense-related biochemicals, suggesting a positive synergistic effect of PAA and pathogen. Furthermore, some metabolites triggered by PAA treatment displayed a similar behavior after *F. oxysporum* infection, suggesting that at least a part of the plant defense responses elicited by PAA mediate same signaling pathways as by this pathogenic fungus. In line with the observation that PAA-induced ISR resembles pathogen-triggered immunity, it can be concluded that PAA primed ISR can be distinguished by the much more pronounced induction in the phenylpropanoid pathway.

## Conclusion

Based on our findings, colonization of *B. fortis* IAGS162 can play a role in suppression of *fusarium* wilt disease by secreting PAA in plant rhizosphere. In our attempts to elucidate the mechanism underlying ISR induced by PAA, we showed that its application extensively re-modulates whole metabolome in tomato plants. This study also provides an insight that phenylpropenoid pathway might be the main factor in this defense process against a fungal pathogen. Undoubtedly, future research on auxins measurement, or the study of auxin-responsive or auxin polar transport genes should be performed to draw a more complete story.

## Author Contributions

WA and TA designed the study. BA drafted the manuscript and critically revised the manuscript. All authors read and approved the final version of the manuscript.

## Conflict of Interest Statement

The authors declare that the research was conducted in the absence of any commercial or financial relationships that could be construed as a potential conflict of interest.

## Footnotes

1 http://mzmine.sourceforge.net/

2 http://biit.cs.ut.ee/clustvis/
